# The urge to breed early: Similar responses to environmental conditions in short‐ and long‐distance migrants during spring migration

**DOI:** 10.1002/ece3.10223

**Published:** 2023-07-04

**Authors:** Georg Rüppel, Ommo Hüppop, Heiko Schmaljohann, Vera Brust

**Affiliations:** ^1^ Institute of Avian Research “Vogelwarte Helgoland” Wilhelmshaven Germany; ^2^ Institute for Biology and Environmental Sciences Carl von Ossietzky University Oldenburg Oldenburg Germany

**Keywords:** bird migration, departure decision, departure timing, multistate model, radio‐telemetry, sea crossing, stopover ecology

## Abstract

Birds migrating different distances experience different temporal, energetic, physiological, and physical constraints throughout migration, which is reflected in their migration strategy. Consequently, we predict different behavioral decisions to similar environmental cues between short‐ and long‐distance migrants, which has been documented for autumn migration. Here, we focus on the question whether trade‐off decisions regarding departure, routing, and landing when alternating between migratory endurance flights and stopovers also differ during spring migration. As early arrivals at the breeding grounds should be ultimately favored regardless of migration distance, selection may favor more similar behavioral decisions in spring than in autumn. We radio‐tagged short‐ and long‐distance migratory songbirds at stopover sites along the German North Sea coast during spring and automatically tracked their migratory behavior using a large‐scale network of receiver stations. Once departed, birds could either cross the sea or detour along the coast. We corrected for spatially biased detection data, using a hierarchical multistate model to assess how birds respond to variation in environmental conditions in their day‐to‐day departure decisions and route selection. The day‐to‐day departure probability was higher in long‐distance migrants independently of the routing decision. Irrespective of migration distance, all species more likely departed under light winds and rainless conditions, while the influence of air pressure change and relative humidity was species‐specific. By accounting for detection probabilities, we estimated that about half of all individuals of each species crossed the sea but did not find differences between short‐ and long‐distance migrants. Offshore flights were more likely when winds blew offshore and began earlier within the night compared with onshore flights. Our results suggest that selection more similarly affects birds of different migration distances in spring than in autumn. These findings put the focus toward how ultimate mechanisms may shape departure and routing decisions differently between migration seasons.

## Graphical abstract

Birds migrating different distances experience different constraints throughout migration, which is reflected in their migration strategy. Consequently, short‐ and long‐distance migrants might show different behavioral decisions to similar environmental cues. Our results suggest that selection more similarly affects short‐ and long‐distance migrants in spring than in autumn, favoring early arrivals at the breeding grounds.

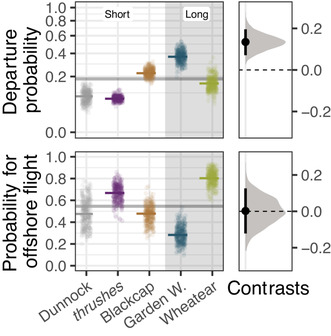

## 1 INTRODUCTION

Avian migrants travel thousands or even tens of thousands of kilometers between their breeding areas and wintering grounds each year (Bairlein et al., 2012; Egevang et al., 2010; Gill et al., 2009). Most of them alternate between migratory endurance flights and periods on the ground, so‐called stopovers (Schmaljohann et al., 2022). Many species mainly migrate during the night (e.g., Alerstam, 2009; Dorka, 1966; Schmaljohann et al., 2007), while they use daytime for resting, recovering, or refueling (e.g., Delingat et al., 2006; Linscott & Senner, 2021; Moore, 2018; Schmaljohann et al., 2022). Therefore, the progress of migration is defined by a sequential series of trade‐off decisions, also called migratory decisions, which include departure, routing, and landing decisions. Departure decisions have two temporal scales, day‐to‐day and within‐night (Müller et al., 2016), and both are directly linked to the routing decision (Schmaljohann & Naef‐Daenzer, 2011). Notably, birds adjust their migratory decisions based on intrinsic factors, for example, energy stores (Deppe et al., 2015; Eikenaar et al., 2016, 2021; Goymann et al., 2010; Schmaljohann et al., 2013) and immune function (Brust, Eikenaar, et al., 2022; Hegemann et al., 2018), and react to extrinsic conditions such as weather (Brust et al., 2019; Klinner & Schmaljohann, 2020; Schmaljohann et al., 2017) and ecological barriers (Deppe et al., 2015; Sandberg & Moore, 1996; Smolinsky et al., 2013). As birds en route constantly react to changing environmental conditions, spatial and temporal migration patterns can be highly flexible among and within individuals (Åkesson & Helm, 2020; Stanley et al., 2012). In particular, migratory decisions shape the spatio‐temporal patterns of individual migration, and thus affect, among others, the birds' distance traveled and energy expended per time unit, and the physiological and physical state (Alerstam, 2001; Nilsson et al., 2013; Schmaljohann et al., 2022). Therefore, studying the proximate reasons for migratory decisions is the key to link migratory decisions to individual fitness. Here, we address the question whether and how migration distances affect the adjustment of migratory decisions on the individual level.

Songbirds are ideal to address this question, as most species, including migration‐naive juveniles, most likely migrate on their own, unguided by conspecifics. The fundamental spatio‐temporal organization of songbird migration is governed by innate circannual clock (Berthold, 1973; Gwinner, 1996) and compass orientation programs (Mouritsen, 2018). These migration programs also define birds' behavioral reactions to variation in intrinsic (e.g., age and sex) (Morbey & Ydenberg, 2001; Schmaljohann et al., 2018) and extrinsic factors (e.g., weather and food) (Schmaljohann et al., 2017; Shamoun‐Baranes et al., 2017).

From a Western Palearctic (WP) perspective, birds performing obligate migrations are commonly categorized as either short‐distance migrants, migrating predominantly within the WP, or trans‐Sahara migrants, migrating between Europe and sub‐Saharan Africa, so‐called long‐distance migrants. Long‐distance migrants likely face stronger time constraints to complete migration. Selection therefore should favor a speed‐maximizing strategy at the expense of relatively high energetic costs (Alerstam, 2011). Short‐distance migrants, in contrast, may benefit from minimizing energy expenditure during migration, since selection for a speed‐maximizing strategy probably is less pronounced (Alerstam, 2011; Nilsson et al., 2014). Packmor et al. (2020) found evidence that individual departure decisions of songbirds during autumn migration depend on migration distance and that short‐ and long‐distance migrants react differently to prevailing environmental conditions at stopover, with long‐distance migrants being least selective at departure. However, the energetic and temporal constraints of migration are context‐dependent and vary within physiological and physical limitations and between species, individuals, but also migration seasons (Schmaljohann et al., 2022). Notably, it remains unclear whether migration distance similarly affects migratory decision adjustment during spring, when early arrivals at the breeding grounds should be mutually beneficial for individual reproductive fitness regardless of the bird's migration distance (Both et al., 2006; Kokko, 1999; Rotics et al., 2018; Smith & Moore, 2005). Therefore, short‐distance as well as long‐distance migrants probably maximize speed of migration at the expense of relatively high risk and energetic costs (Alerstam, 2011), leading to more similar behavioral decisions between them in spring than in autumn.

To fill parts of this gap in knowledge, we studied departure and routing decisions in songbirds using miniature radio‐transmitters within a large‐scale network of automated radio‐receiving stations (Taylor et al., 2017), since heavier tags required for more accurate GPS‐tracking are not feasible for small songbirds (Bridge et al., 2011). However, the challenge with radio‐tracking is that the spatial coverage is limited by the array of receiving stations. Since they are usually non‐equally distributed, observation heterogeneity often leads to spatially biased detection data. Ultimately, this bias can result in false interpretations of causal inference regarding movement decisions. Hierarchical multistate models can help to overcome these issues by explicitly modeling the observation process (Calvert et al., 2009; Hooten et al., 2017). This method specifically models the underlying individual behavioral process of a time series of movement steps (Schick et al., 2008). We used this discrete latent state process to model sequential series of migratory decisions, that is, day‐to‐day departure and routing decisions. This approach makes the data conditionally dependent on the true movement observed with error, that is, different detection probabilities.

Following this approach, we asked (i) whether departure (day‐to‐day and within‐night) and routing decisions differ between short‐ and long‐distance migrants during spring, and (ii) whether these departure decisions differ with respect to the prevailing weather conditions between the migration groups.

## 2 METHODS

### 2.1 Study sites and species

**FIGURE 1:**
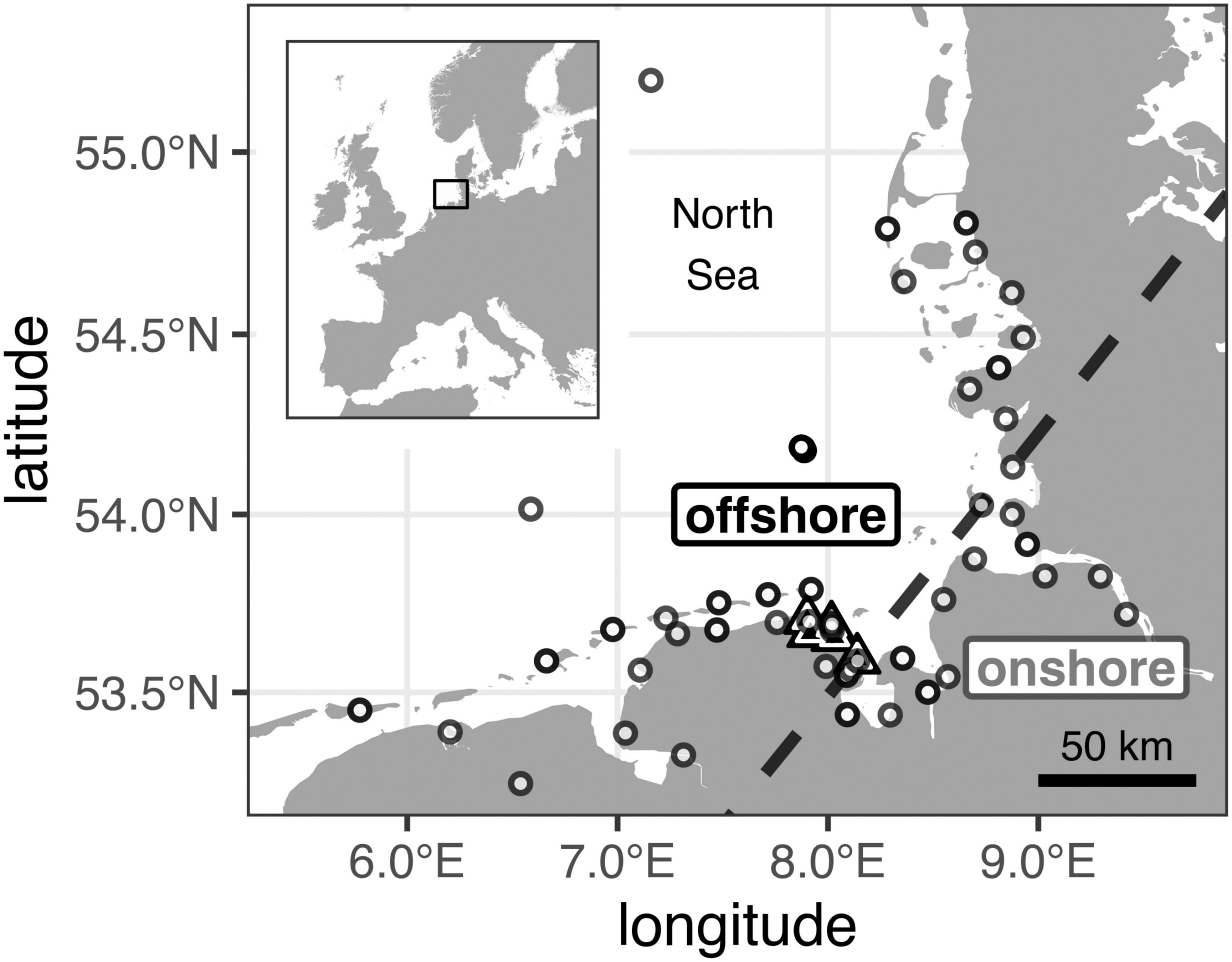
Study area with locations of tag deployment (triangles) and receiver stations (circles). The dashed black line indicates the threshold latitude and longitude for flight categorization as offshore (to the west) or onshore (to the east) flight.

We caught songbirds with mist nets at the German North Sea coast of Lower Saxony (Figure 1) during spring migration. In total, we caught 289 birds across seven species, that is, the short‐distance migrants Dunnock *Prunella modularis*, Eurasian Blackbird *Turdus merula*, Song Thrush *Turdus philomelos*, Redwing *Turdus iliacus*, and Blackcap *Sylvia atricapilla* and the long‐distance migrants Garden Warbler *Sylvia borin*, and Northern Wheatear *Oenanthe o. oenanthe* (Table 1). Catching was mainly conducted in 2018 and 2020 with catching periods ranging from 13 March to 17 May. Additionally, nine and 14 *oenanthe* Northern Wheatears were caught in 2017 and 2019, respectively (Brust et al., 2023; Appendix S1: S1). Due to the small sample sizes in Song Thrushes (*n* = 17), Redwings (*n* = 11), and Eurasian Blackbirds (*n* = 25), their widely overlapping breeding areas and similar migration ecology, we pooled these species as “thrushes” (cf. Brust et al., 2019; Dierschke et al., 2011).

**TABLE 1:** Number of observed departure states and total number of radio‐tagged individuals per species.

Species	Departure state			Not detected	Tagged
	Offshore	Onshore	Departed^a^		
**Short‐distance migrants**					
Dunnock *Prunella modularis*	7	8	1	17	33
Eurasian Blackbird *Turdus merula*	9	6	10	10	35
Song Thrush *Turdus philomelos*	3	6	8	12	29
Redwing *Turdus iliacus*	0	3	8	8	19
Blackcap *Sylvia atricapilla*	11	23	15	33	82
**Long‐distance migrants**					
Garden Warbler *Sylvia borin*	7	27	4	13	51
Northern Wheatear *Oenanthe o. oenanthe*	12	4	12	12	40
Total	49	77	58	105	289

^a^ Based on detection data at stopover, routing unknown.

### 2.2 Radio‐tracking

Immediately after catching, birds were fitted with uniquely coded radio‐transmitters using leg‐loop harnesses adjusted to body size (Naef‐Daenzer, 2007). For thrushes, we used transmitters of type ACT‐521, the smaller species were equipped with lighter tags of types NTQB‐1, NTQB2‐1, or NTQB2‐2 (Lotek Wireless Inc.) with less transmitting power (Appendix S1: S2). The mass of radio‐transmitters including harness did not exceed 2.5% of the body masses of the birds at capture (Appendix S1: S3). Birds were immediately released at the catching site and tracked by an automated radio‐telemetry receiver network that covers much of the coastline and islands along the German Bight (Figure 1). All receiver stations are part of the global collaborative Motus Wildlife Tracking System (Taylor et al., 2017); for more information, see http://www.motus.org.

Detection data were processed by and retrieved from Motus using the motus R package (Brzustowski & LePage, 2021) and presumably false positives were discarded following the filtering routine described in Brust et al. (2019). We identified continuous movements (hereafter referred to as “flights”) within the individual tracking data. Following Brust et al. (2019), a movement was defined as flight when it covered a distance of at least 35 km or was recorded by a minimum of three different receivers with consecutive detections in <7 h. In most cases, birds passed by all including the last receiver station with detections of that bird, indicating that they were still in flight, and the data represent only portions of longer flight bouts. Three individuals were excluded from the analysis due to exceptionally long stopover durations of more than 40 days (for minimum stopover durations, see Appendix S2: S5), suggesting that these birds were probably local breeders. We did not include five short flights along the coast heading west against the main migration direction in spring, since these very likely do not refer to migratory endurance flights, but to landscape movements or similar behavior during stopover (Michalik et al., 2020; Mills et al., 2011; Schmaljohann & Eikenaar, 2017; Taylor et al., 2011). For seven birds, more than one flight was detected, but only the first flight of each individual was included in the subsequent analyses to avoid pseudoreplication. These birds showed multiple detections but then stopped somewhere and had a second flight.

We assigned each flight to the departure states “offshore” or “onshore.” An offshore‐oriented flight began at geographic longitudes west of 8.08°E and ended at geographic latitudes north of 54.135°N or included detections at an offshore receiver station such as Helgoland or FINO3 (Brust et al., 2019, Figure 1).

Among the individuals for which no flight was detected, we identified departures based on the latest receiver detection data available from the stopover site (<20 km from the location of tag deployment). Departure events show distinct patterns of detected signal strength (Appendix S1: S4). We therefore assumed a departure if the mean of the last five detections of a single antenna was below the mean of the highest signal strength within the last 10 min prior to signal loss and its four neighboring values (for more details, see Appendix S1: S4). We did not include two departures of Blackcaps and five flights of Garden Warblers, which occurred immediately after tag deployment in the morning. Given that both species are typical nocturnal migrants (Dorka, 1966), such movements may also refer to stopover behavior, for example, landscape movements (see above). For 105 birds, departure times could not be determined by either method. These individuals were not included in any analysis as they may have lost their tags, died from predation, or the stopover duration exceeded the battery lifetime of the tags.

Experiments were conducted with the approval of the Lower Saxony State Office for Consumer Protection and Food Safety (LAVES) in Lower Saxony (license number 33.19‐42,502‐04‐16/2349).

### 2.3 Weather data

Hourly weather data with a spatial resolution of 0.25° × 0.25° were obtained from ERA5 reanalysis accessed via the Copernicus Climate Change Service (Hersbach et al., 2018a, 2018b; Muñoz‐Sabater et al., 2021), including eastward and northward wind components, air pressure, temperature, and relative humidity. Since songbird migration in coastal central Europe is concentrated at low elevations, that is, 0–1500 m above sea level (Bruderer et al., 2018; Hüppop et al., 2006), all parameter values applied to near‐surface level (≤10 m). Data were fetched individually from the respective grid cell to the location where each flight began and for the time of each sunset (rounded to the full hour) until the departure of the respective bird. Furthermore, we calculated the change in air pressure and temperature as the difference between the measurement at each sunset and the respective measurement 24 h before. We also included hourly precipitation data (binary index: rain vs. no rain) for the time of each sunset from the weather station Wangerland‐Hooksiel (53.64°N, 8.08°E) accessed via the Open Data Server of the German Weather Service (DWD Climate Data Center, 2021, 2022).

### 2.4 Statistics

We run linear multilevel models to estimate temporal differences in departure decisions between both routing decisions, that is, offshore and onshore flights, and species, including their interaction. For all birds with known routing, we first estimated the minimum stopover duration in days using a negative binomial regression to account for overdispersion, which was present in our data (Appendix S2: S5). In a second model, we estimated the time of departure relative to night length using a Student‐*t* linear regression (Appendix S2: S6).

To assess weather effects on departure and routing decisions, we formulated a multistate capture–recapture model (Kéry & Schaub, 2012; Lebreton et al., 2009), that is, a hidden Markov model with unsupervised parameter estimation (Schick et al., 2008; Stan Development Team, 2022). In this four‐state model, all birds initially were in the state “stopover” at tag deployment. In case of a detected flight, the state of a bird then changed to the departure state of that individual, that is, “offshore” or “onshore,” assuming unbiased classification. The resulting state‐transition matrix (Ω) and the observation matrix (Θ) are

(1) $$\Omega =\left[\begin{array}{cccc} 1-\psi & \psi \chi & \psi \left(1-\chi \right) & 0\\ 0 & 0 & 0 & 1\\ 0 & 0 & 0 & 1\\ 0 & 0 & 0 & 1 \end{array}\right],\Theta =\left[\begin{array}{cccc} 1 & 0 & 0 & 0\\ 0 & \mathrm{pX} & 0 & 1-\mathrm{pX}\\ 0 & 0 & \mathrm{pC} & 1-\mathrm{pC}\\ 0 & 0 & 0 & 1 \end{array}\right],$$

where ψ is the day‐to‐day departure probability, χ is the probability for an offshore flight, and pX and pC are the detection probabilities for offshore and onshore flights, respectively. The states (*z*, from top to bottom) are “stopover,” “offshore,” “onshore,” and “departed.” The encounter history matrix *y* contained 184 individuals (*i*) and 36 occasions (*t*). The following equations define the state and observation processes, respectively:

(2) $$z_{i,t+1}|z_{i,t}∼\text{Categorical}\left(\Omega_{z_{i,t},1\ldots S,i,t}\right)$$

(3) $$y_{i,t}|z_{i,t}∼\text{Categorical}\left(\Theta_{z_{i,t},1\ldots O,i,t}\right)$$

(4) $$z_{i,1}=1$$

We estimated the day‐to‐day departure probability for each individual ($\psi_{i,t}$) as a function of a species‐specific intercept ($\beta_{0,s}^{\psi }$), quadratic species‐specific relationships with both wind components using orthogonal polynomials ($u_{l\left[i,t\right]}$, $u_{q\left[i,t\right]}$, $v_{l\left[i,t\right]}$, $v_{q\left[i,t\right]}$) allowing lower departure probabilities in strong winds, linear species‐specific relationships with pressure change ($\Delta p_{i,t}$) and relative humidity ($h_{i,t}$), and a linear relationship with the binary precipitation indicator ($r_{i,t}$):

(5) $$\text{logit}\left(\psi_{i,t}\right)=\beta_{0,s}^{\psi }+\beta_{1,s}^{\psi }u_{l\left[i,t\right]}+\beta_{2,s}^{\psi }u_{q\left[i,t\right]}+\beta_{3,s}^{\psi }v_{l\left[i,t\right]}+\beta_{4,s}^{\psi }v_{q\left[i,t\right]}+\beta_{5,s}^{\psi }\Delta p_{i,t}+\beta_{6,s}^{\psi }h_{i,t}+\beta_{7}^{\psi }r_{i,t}$$

An interaction between precipitation and species was not included, as there were relatively few days with rain in our data (10 days out of 128). We used a similar approach to model the probability for an offshore flight ($\chi_{i,t}$), but did not include interactions with species, due to low sample size:

(6) $$\text{logit}\left(\chi_{i,t}\right)=\beta_{0,s}^{\chi }+\beta_{1}^{\chi }u_{l\left[i,t\right]}+\beta_{2}^{\chi }u_{q\left[i,t\right]}+\beta_{3}^{\chi }v_{l\left[i,t\right]}+\beta_{4}^{\chi }v_{q\left[i,t\right]}$$

Since sampling periods within years concentrated on the main passage times of the respective species in northern Germany (Dierschke et al., 2011), we did not estimate effects of the relative departure date within season on migratory decisions. Tag‐specific detection probabilities, that is, “ACT” versus “NTQB” tags, were modeled constantly over time. Since no actual measurements of actual transmitting power were available, the only prior information we added was that the power and thus the detection probability of “ACT” tags should be higher compared to “NTQB” tags. All continuous explanatory variables were centered and scaled to one standard deviation (SD) prior to analysis.

All models were fitted using the Hamiltonian Monte Carlo engine Stan (Carpenter et al., 2017) in R 4.2.1 (R Core Team, 2022) from CmdStanR 0.5.3 (Gabry & Češnovar, 2022). Priors were weakly informative with minimal influence on the result. We sampled using four MCMC chains for 2000 iterations, including 1000 adaptation steps. All model parameters showed successful convergence and inferences are based on over 1000 effective samples from the posterior (for details, see Appendix S2). Model adequacy was assessed by graphical posterior predictive checks (Gelman et al., 2013). We report posterior distribution means and 90% highest posterior density intervals (HPDI).

## 3 RESULTS

In total, we analyzed data of 126 individual flights (76 short‐ and 50 long‐distance migrants, 49 offshore and 77 onshore flights) combined with 58 individual departures for which no flights were recorded (Table 1). Estimation of temporal differences in departure decisions between both routing decisions is based on the 126 individuals with known routing.

### 3.1 Departure

**FIGURE 2:**
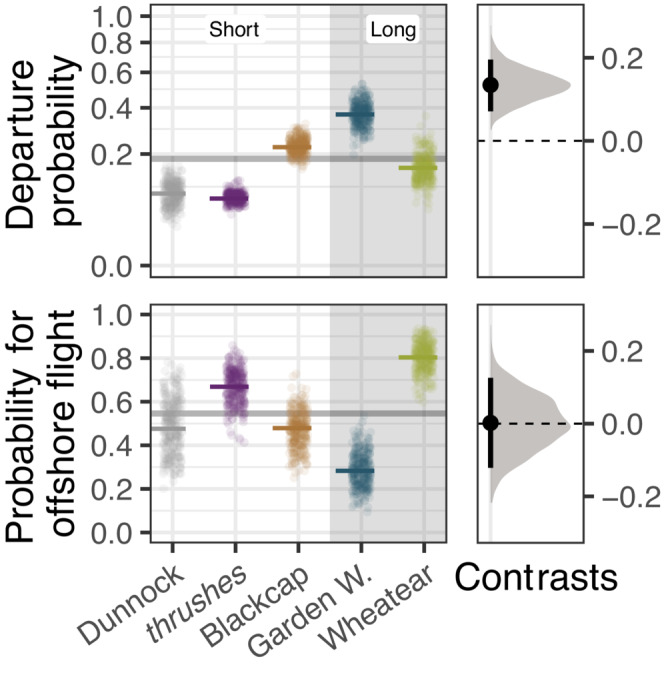
Mean departure probability and mean probability for offshore flights per species (*Turdus* species are pooled) and migration distance (short: short‐distance migrants, long: long‐distance migrants) during spring. Estimates (dashes) are given together with a subset of 300 draws from the joint posterior distribution. The overall means are indicated by horizontal lines. The right panels show posterior distributions for respective contrasts between long‐ and short‐distance migrants together with means (dots) and 90% highest posterior density intervals (bars).

**FIGURE 3:**
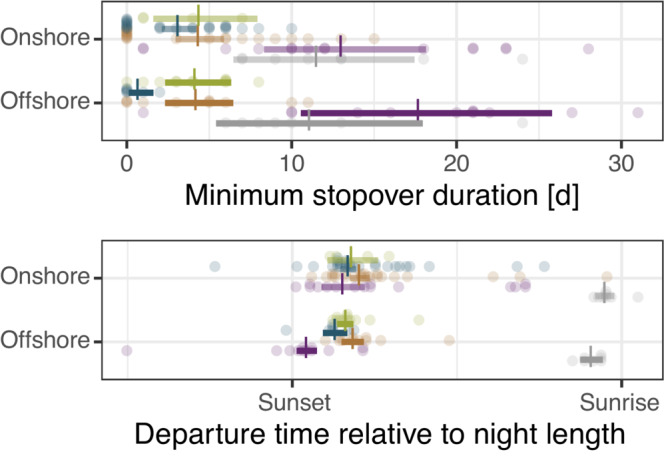
Variations in minimum stopover duration and departure time relative to night length per departure state and species (*Turdus* species are pooled). Estimates (dashes) and 90% highest posterior density intervals (bars) are given together with the observed raw data.

**FIGURE 4:**
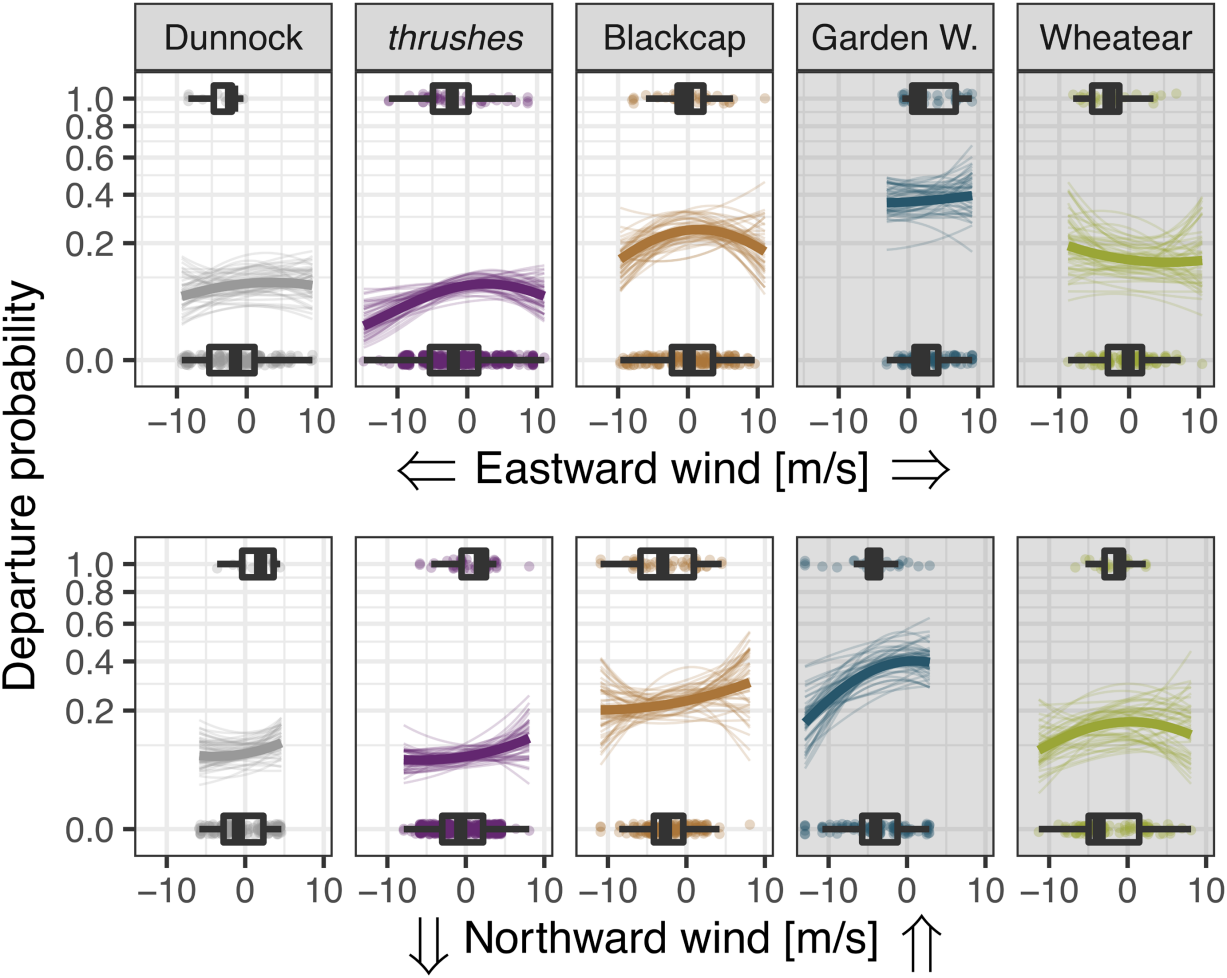
Influence of wind on departure decision, that is, the day‐to‐day departure probability, from stopover during spring. Negative values correspond to westward and southward wind, respectively. Species‐specific predictions (thick lines) and a subset of 50 draws from the joint posterior distribution (thin lines) are given together with the observed raw data (dots, boxplots). *Turdus* species are pooled. Predictions were made for rainless conditions with the remaining model covariates set to their means. Note the square‐root scale of the *y*‐axis.

**FIGURE 5:**
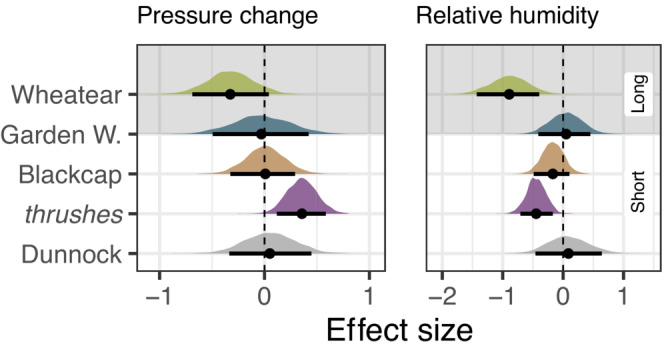
Influence of air pressure change and relative humidity on departure decision, that is, the day‐to‐day departure probability, from stopover per species (*Turdus* species are pooled) during spring (short: short‐distance migrants, long: long‐distance migrants). Posterior densities of the estimates are given per species together with means (dots) and 90% highest posterior density intervals (bars).

The mean day‐to‐day departure probability over all species was 18.3% (90% HPDI: 15.4%–20.8%), ranging from 7.4% (5.7%–9.2%) in thrushes to 37.4% (28.6%–46%) in Garden Warblers (Figure 2). Mean stopover duration of short‐distance migrants was 10.7 days (8.2–13 days) and on average 7.4 days (4.7–10.1 days) longer than the mean stopover duration of long‐distance migrants, which was 3.3 days (2.2–4.4 days; Figure 3). Within‐night departure decision differed between species, with most birds departing within the first half of the night while all Dunnocks departed around sunrise (Figure 3, Appendix S2: S6). Onshore flights began 4.6% of night length (1.9%–7.4%) later compared to offshore flights (Figure 3), which is a difference of about half an hour given a mean night length of 9.3 ± 1 h (mean ± SD, *n* = 128). All species more likely departed under eastward winds (westward winds in Northern Wheatears), light northward winds (Figure 4), and dry conditions (Appendix S2: S7). The influence of air pressure change and low relative humidity differed between species but not between short‐ and long‐distance migrants. While pressure change showed a weak and inconsistent influence on the departure decision among species, departure probability consistently decreased under lower relative humidity in some species (Figure 5). For detailed information on model results per parameter, see Appendix S2.

### 3.2 Routing

**FIGURE 6:**
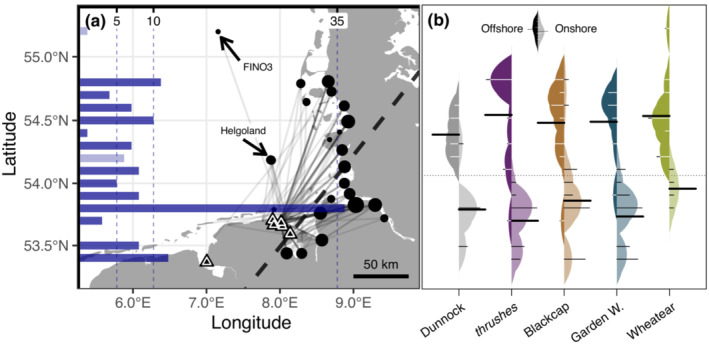
Final detection latitudes of songbirds after leaving coastal stopover sites in north‐western Germany. (a) Map indicating locations of tag deployment (triangles) and receiver stations where birds were finally detected after a migratory endurance flight (dots, size equals to the number of individuals). The histogram on the left summarizes the number of individuals detected per 0.1°. Offshore detections on Helgoland and FINO3 are given in light colors. The dashed black line indicates the threshold latitude and longitude for flight categorization as offshore (to the left) or onshore (to the right) flight. (b) Bean plots of final detection latitudes per species (*Turdus* species are pooled) and departure state. Extensions of the bean plots represent kernel density estimations of the distribution of individual latitudes, given as small lines in the plot. The broad lines represent the mean latitudes of the final detection per group, and the overall average is given by a dashed line.

**FIGURE 7:**
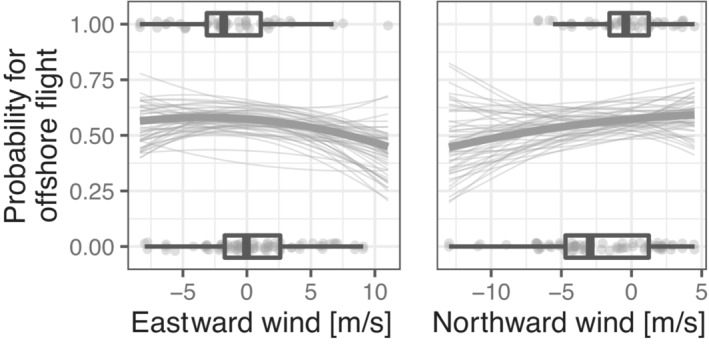
Influence of wind on routing decision, that is, offshore or onshore flights, during spring. Predictions (thick lines) and a subset of 50 draws from the joint posterior distribution (thin lines) are given together with the observed raw data (dots, boxplots). Predictions were made with the remaining model covariates set to their means.

We estimated a mean number of 99 (83–113) offshore flights, resulting in a proportion of 53.8% (45.1%–61.4%). The mean latitude of the final detection was 54.48°N ± 0.31° for offshore flights and 53.80°N ± 0.26° for flights along the coast (Figure 6). We did not find differences in the probability for offshore flights between short‐ and long‐distance migrants (Figure 2). The Garden Warblers had the lowest mean probability for offshore flights (29.1%, 15.3%–41.4%), while the highest probability was found in the Northern Wheatear (80.9%, 69.2%–94.2%). Routing was predicted by both wind components with higher probabilities for an offshore flight under westward and northward (offshore) winds peaking at low wind speeds (Figure 7). For detailed information on model results per parameter, see Appendix S2.

## 4 DISCUSSION

Here, we show that departure probability from a stopover site is related to migration distance during spring, but, in contrast to the study by Packmor et al. (2020) in autumn, found no consistent differences in reactions to the prevailing environmental conditions between five short‐ and two long‐distance migrants. Depending on the temporal, energetic, physiological, and physical constraints encountered during migration, we expect individual migratory decisions to be well adapted to minimize the costs of migration. We therefore hypothesize that seasonal patterns in migratory decisions may arise from selection acting more similarly on short‐ and long‐distance migrants during spring in favor of early arrivals at the breeding grounds.

### 4.1 Departure

#### 4.1.1 Departure timing

The time required to complete migration is determined mainly by stopover duration and less by flight speed (Nilsson et al., 2013; Schmaljohann, 2018), which makes the total stopover duration an important factor influencing the arrival time at the migratory destination (Alerstam, 2011; Schmaljohann & Both, 2017). Therefore, the individual departure decisions at stopover to minimize temporal or energetic constraints may reflect to a certain extent the respective species‐ or population‐specific migration distance (Packmor et al., 2020). We found shorter stopovers, that is, higher day‐to‐day departure probability, in long‐distance migrants compared with short‐distance migrants (Figure 2), which generally supports the findings of Packmor et al. (2020). Furthermore, shorter stopovers in long‐distance migrants are consistent with present evidence that the total speed of migration increases with migration distance, especially during spring (La Sorte et al., 2013; Schmaljohann, 2019). As the passage of long‐distance migrants in central Europe is phenologically shifted toward the end of spring migration compared with short‐distance migrants (Hüppop & Hüppop, 2011), the following context‐dependent effects, rather than migration distance itself, may possibly explain specific patterns in departure decision. First, increasing food availability later in the season may enable a higher fuel deposition rate (Lindström et al., 2019), allowing shorter stopovers (Alerstam & Lindström, 1990; Jonzén et al., 2007; Lindström & Alerstam, 1992). Second, long‐distance migrants in our study experienced on average 1.8 h (1.7–2 h) shorter nights compared with short‐distance migrants. Therefore, more nights with migratory endurance flights and hence more individual departure events are required to reach the same potential flight duration (Müller et al., 2016). Although there was some overlap in departure probabilities among different species of short‐ and long‐distance migrants, we found good evidence that migration distance may be of general importance for the day‐to‐day departure decision in spring. However, since the number of species considered by Packmor et al. (2020) and in the present study is low, further research on different species is required for conclusive assessment.

Nocturnal migrants usually terminate migratory endurance flights before sunrise (Dorka, 1966; Liechti et al., 2018; Müller et al., 2016). When leaving a stopover for a migratory endurance flight under favorable environmental conditions and with sufficient energy stores, birds therefore should depart early within the night to maximize the time available to complete the respective flight bout (Alerstam, 2009; Müller et al., 2016, 2018). We found that birds that flew offshore departed on average half an hour earlier within the night/day compared with individuals that followed the coast (Figure 3). By doing so, birds optimize the time available to cross the sea under favorable flight conditions (Alerstam, 2009) and save approximately 50 km flight distance or 1 h of flight in still air. Late departures in the night/day may refer to migratory endurance flights or broad‐scale landscape movements (Mills et al., 2011; Taylor et al., 2011). The latter probably involves relocations in search of suitable habitats and thus does not represent migratory flights in the strict sense (Schmaljohann & Eikenaar, 2017; Taylor et al., 2011).

#### 4.1.2 Departure and weather

We found that short‐ and long‐distance migrants similarly adjusted individual departure decisions in respect of prevailing wind conditions. Most birds more likely departed under tailwind conditions (Figure 4) given the predominant north‐east orientation of spring migration in central Europe (Nussbaumer et al., 2022), including the North Sea area (Dierschke et al., 2011). A marked difference in Northern Wheatears might be due to the fact that the proportion of birds oriented to breeding grounds further north, or even further west, for example, on the British Isles, is considerably higher compared with the other species (Brust et al., 2023; Delingat et al., 2011; Keller et al., 2020). Our results thus support the notion that wind affects the birds migratory behavior, including departure decision (see also e.g., Brust et al., 2019; Deppe et al., 2015; Dossman et al., 2016; Schmaljohann & Naef‐Daenzer, 2011; Sjöberg et al., 2017), probably through its effect on energy expenditure and time budget during flight (Liechti, 2006; Shamoun‐Baranes et al., 2017). In our study, departure probability decreased during rain (Appendix S2: S7), which supports previous findings (Newton, 2007; Schaub et al., 2004). Notably, the influence of air pressure change and low relative humidity differed among species but not between short‐ and long‐distance migrants (Figure 5). Increases in air pressure and low humidity are indicative of beneficial weather and therefore provide potential cues to predict favorable flight conditions in the near term (Åkesson et al., 2002; Metcalfe et al., 2013; Richardson, 1990; Schmaljohann et al., 2017). Therefore, the influence of these cues on departure decision may be more moderate and thus weaker and more inconsistent than of weather conditions that directly affect energy expenditure like wind and rain.

### 4.2 Routing

Given that there are very few offshore receiver stations compared to the coastline, presumably more onshore than offshore flights are detected via radio‐telemetry. By explicitly modeling the underlying observation process, our approach enabled us to account for such differences in detection probabilities. In this way, hierarchical modeling can help to correct for biased detection data in radio‐telemetry due to heterogeneously distributed receiver stations. We estimated that about half of the observed individuals crossed the German Bight by flying over open waters, while the probability to detect an onshore flight was around 95%, compared with only 50% for offshore flights (Appendix S2: S7).

Although the mean probability for offshore flights varied among species, we found no consistent differences between short‐ and long‐distance migrants (Figure 2). Therefore, we expect migrating songbirds to cross the German Bight throughout the migration season, even though the species composition of short‐ and long‐distance migrants changes over time. Not surprisingly, the probability for offshore flights increased with winds blowing offshore (Figure 7). Nevertheless, migratory endurance flights over ecological barriers should be minimized when birds do not benefit from wind assistance (Alerstam, 2001). With increasing cross wind, birds are drifted away from the migratory destination (Liechti, 2006). Notably, our data revealed some indication of adjusted flight bearings to mitigate wind drift in order to compensate for substantial displacement under such conditions (Appendix S2: S9). In particular, individuals of the same species took contrary routing decisions within the same night when experiencing the same environmental conditions, confirming that individual routing decisions are a particularly flexible and complex part of migration (Åkesson & Helm, 2020; Brust et al., 2019; Stanley et al., 2012). Part of the individual variation is probably explained by different intrinsic conditions, for example, energy stores (Deppe et al., 2015; Müller et al., 2018, Appendix S2: S8) and health status (Brust, Eikenaar, et al., 2022; Hegemann et al., 2018).

## 5 CONCLUSION

Migration in songbirds is mainly genetically controlled (Gwinner, 1996), while energetic and temporal constraints are context‐dependent (Schmaljohann et al., 2022). Here we propose that, despite high individual en route flexibility, selection more similarly affects birds with different migration distances during spring than in autumn in favor of early arrivals at the breeding grounds. These findings put the focus on how ultimate mechanisms may shape departure and routing decisions differently between spring and autumn.

## AUTHOR CONTRIBUTIONS

**Georg Rüppel:** Formal analysis (equal); methodology (equal); writing – original draft (equal). **Ommo Hüppop:** Conceptualization (equal); funding acquisition (equal); resources (equal); writing – review and editing (equal). **Heiko Schmaljohann:** Funding acquisition (equal); resources (equal); writing – review and editing (equal). **Vera Brust:** Conceptualization (equal); investigation (equal); resources (equal); writing – review and editing (equal).

## CONFLICT OF INTEREST STATEMENT

We declare that we have no competing interests.

## Supporting information


Appendix S1

Appendix S2

## Data Availability

All data and materials have been made publicly available and can be accessed via GitHub at https://github.com/g‐rppl/spring_migDist and the Zenodo archiv https://doi.org/10.5281/zenodo.8026843.
