# Supplementary material for: The urge to breed early: Similar responses to environmental conditions in short‐ and long‐distance migrants during spring migration
Source: Ecol Evol. 2023 Jul 4;13(7):e10223. doi: 10.1002/ece3.10223 (PMC10318620; doi:10.1002/ece3.10223)
Supplement: Supplementary file 1 — Appendix S1 [file ECE3-13-e10223-s002.pdf]

The urge to breed early: Similar responses to environmental conditions in short- and long-distance migrants during spring migration

Supporting Information: Telemetry

Georg Rüppel, Ommo Hüppop, Heiko Schmaljohann & Vera Brust

S1 Timing of fieldwork

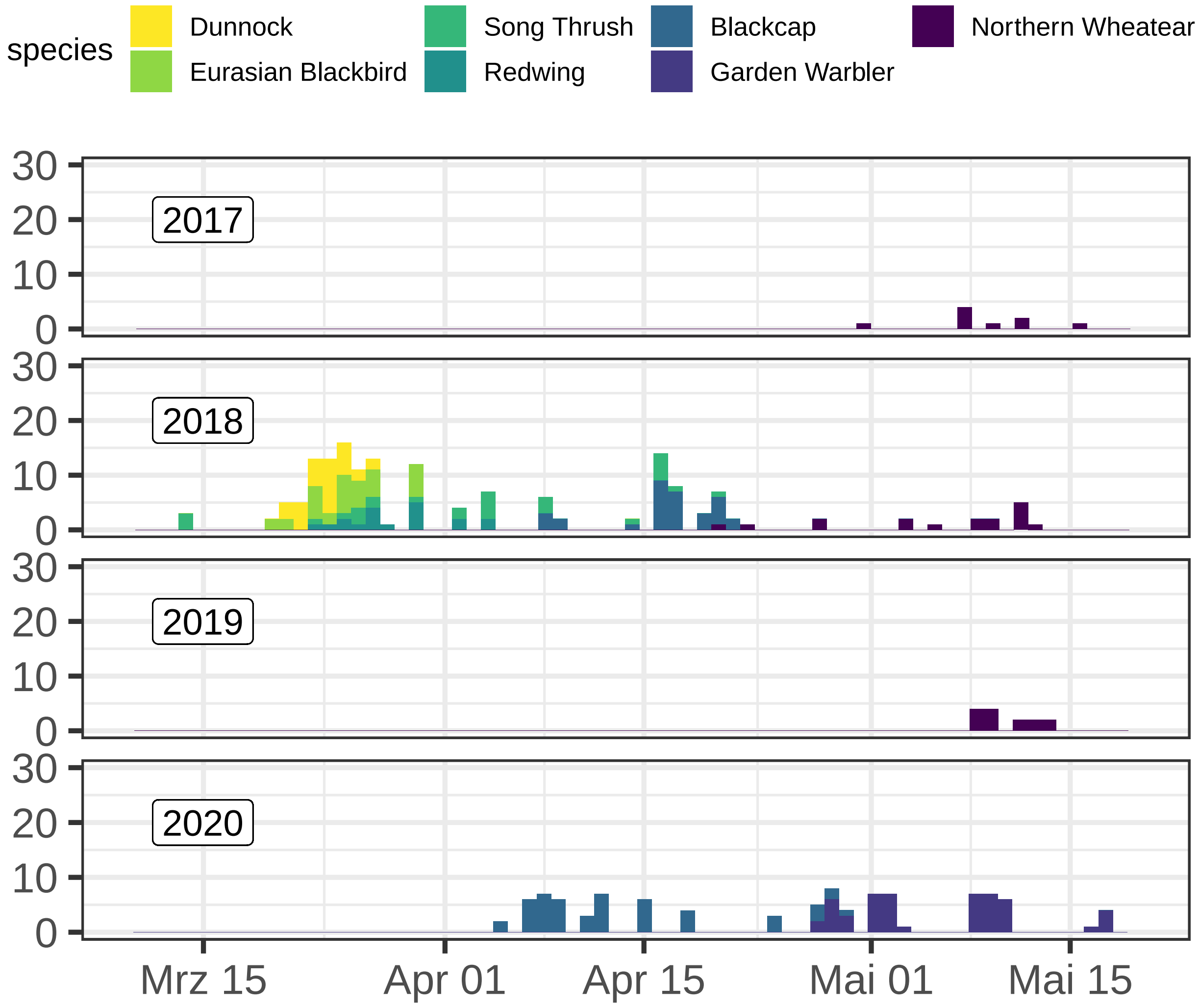

Figure S1: Number of radio-tagged birds per species during the study period.

S2 Tag types

Table S1: Number of tag types used per species.

	ACT-521	NTQB-1	NTQB2-1	NTQB2-2
Dunnock	0	0	33	0
Eurasian Blackbird	35	0	0	0
Song Thrush	29	0	0	0
Redwing	17	0	2	0
Blackcap	0	0	82	0
Garden Warbler	0	0	51	0
Northern Wheatear	0	9	17	14

S3 Tag weights and body masses

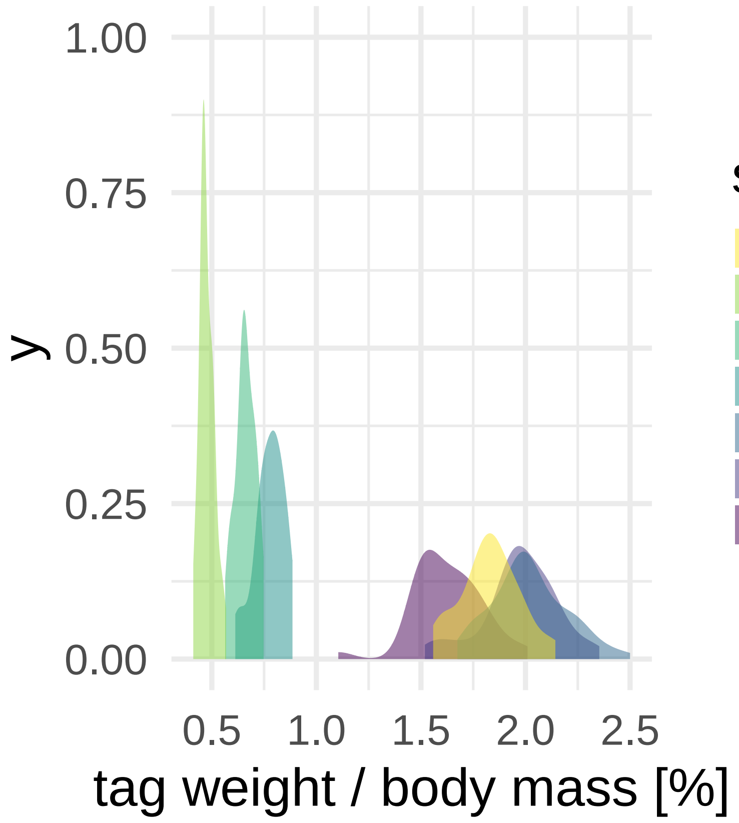

Figure S2: Species-specific distributions of tag weights (including 0.1 g harness) relative to individual body masses.

S4 Identify departures from detection data

If a bird leaves a stopover site from within the range of a receiver station, the detected signal strength rapidly increases when the bird takes of and then decreases continuously as the bird moves away from the antenna until the signal is lost. In case of a fly-by signal strength changes continuously and finally decreases as the bird moves away from the antenna. In either case the signal strength of the latest detections is lower than the detected maximum signal strength of the run. Thus, to identify departure events based on detection data we looked at the last ten minutes of raw signal strength data per individual (i) and antenna (a). We calculated the mean of the last five signals per antenna ($\mu_{end[i,a]}$) and compared it to the mean of the signal with the maximum signal strength and its four neighbouring values ($\mu_{max[i,a]}$). If at least one signal strength $\mu_{end[i,a]}$ was below $\mu_{max[i,a]}$ we assumed a departure of the individual at this specific day.

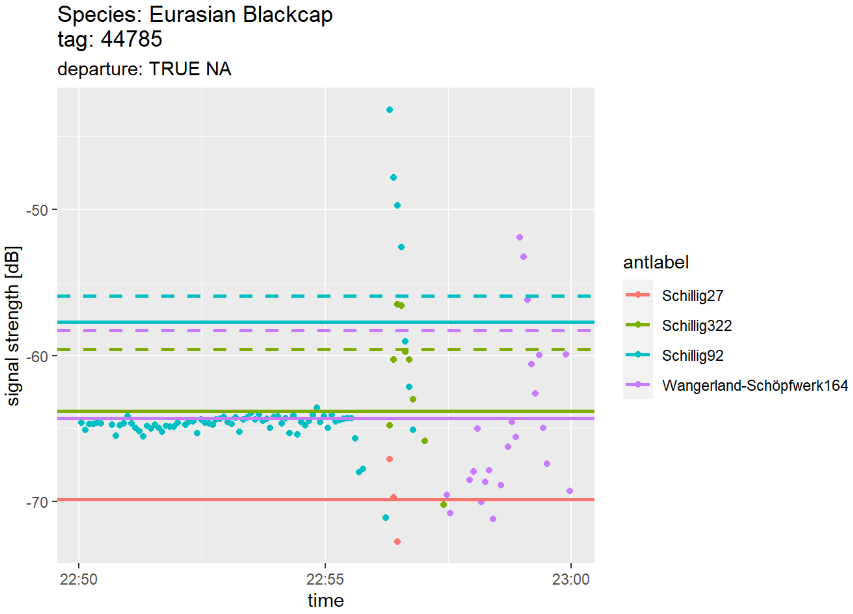

Figure S3: Departure event as recorded by the automated radiotelemetry system showing raw signal strength data against time (UTC). Colours denote signals received by different antennas at two receiving stations. The means of the last five signals per antenna ($\mu_{end_{i,a}}$) are given as solid lines, the means of the maximum value and its four neighboring values ($\mu_{max_{i,a}}$) are given as dashed lines.
