# Supplementary material for: The urge to breed early: Similar responses to environmental conditions in short‐ and long‐distance migrants during spring migration
Source: Ecol Evol. 2023 Jul 4;13(7):e10223. doi: 10.1002/ece3.10223 (PMC10318620; doi:10.1002/ece3.10223)
Supplement: Supplementary file 2 — Appendix S2 [file ECE3-13-e10223-s001.pdf]

The urge to breed early: Similar responses to environmental conditions in short- and long-distance migrants during spring migration

Supporting Information: Modelling

Georg Rüppel, Ommo Hüppop, Heiko Schmaljohann & Vera Brust

S5 Minimum stopover duration

S5.1 Raw data

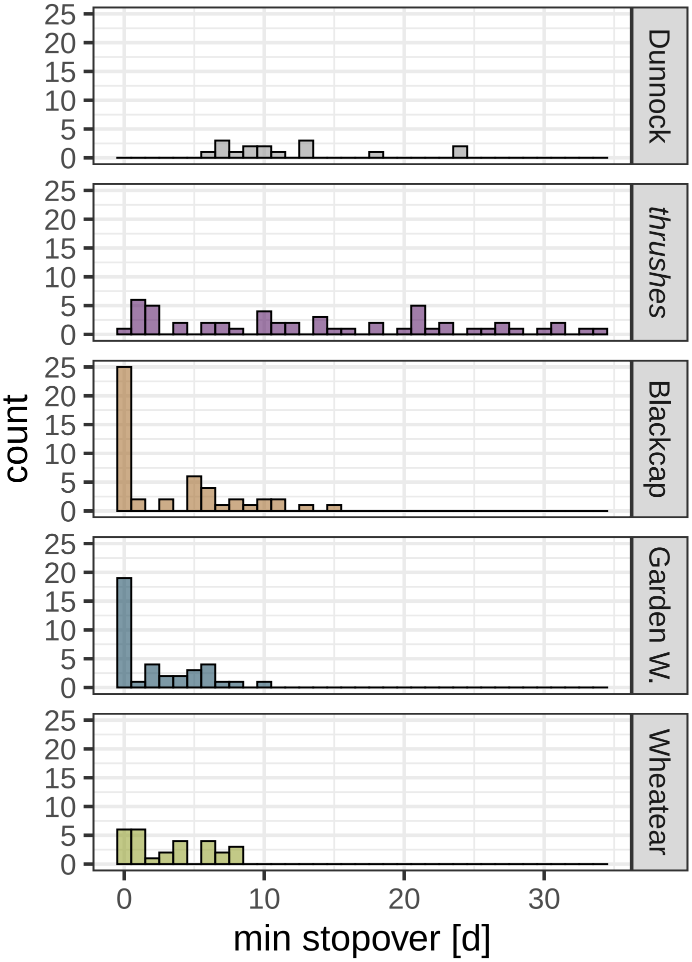

Figure S4: Variation in minimum stopover durations at the initial stopover site, i.e. days between tag deployment and detected departure, per species (*Turdus* species are pooled).

S5.2 Model formulation

$$
\begin{aligned}
y_i &\sim \text{Negative binomial}(\lambda_i, \phi) \\
log(\lambda_i) &= \beta_{sp[i],st[i]} \\
\begin{bmatrix} \beta_{j,1} \\ \beta_{j,2} \end{bmatrix} &\sim \text{MVNormal}\left(\begin{bmatrix} \mu_1 \\ \mu_2 \end{bmatrix}, S_{sp} \right) \\
\mu_1 &\sim \text{Normal}(\log(7), 2) \\
\mu_2 &\sim \text{Normal}(\log(7), 2) \\
S_{sp} &= \begin{pmatrix} \sigma_{\mu[1]} & 0 \\ 0 & \sigma_{\mu[2]} \end{pmatrix} R \begin{pmatrix} \sigma_{\mu[1]} & 0 \\ 0 & \sigma_{\mu[2]} \end{pmatrix} \\
\sigma_{\mu[1]} &\sim \text{Exponential}(0.5) \\
\sigma_{\mu[2]} &\sim \text{Exponential}(0.5) \\
R &\sim \text{LKJcorr}(2) \\
\phi &\sim \text{Exponential}(1)
\end{aligned}
$$

S5.3 Model code

```
data{
  int<lower=0> Nind; // number of individuals
  int<lower=0>  Nsp; // number of species
  array[Nind] int<lower=0> y; // min stopover duration
  array[Nind] int<lower=1, upper=Nsp> sp; // species index
  array[Nind] int<lower=1, upper=2>   st; // state index
}

parameters{
  vector[2] mu; // group-level mean
  array[Nsp] vector[2] b; // beta
  corr_matrix[2] Rho; // correlation matrix
  vector<lower=0>[2] sigma; // group-level SD
  real<lower=0>  phi; // scaling parameter
}

model{
  vector[Nind] lambda;

  // Priors and constraints
  mu    ~ normal(log(7), 2);
  b     ~ multi_normal(mu, quad_form_diag(Rho, sigma));
  Rho   ~ lkj_corr(2);
  sigma ~ exponential(0.5);
  phi   ~ exponential(1);
  for (i in 1:Nind) {
    lambda[i] = b[sp[i], st[i]];
  }

  // Likelihood
  target += neg_binomial_2_log_lpmf(y | lambda, phi);
}

generated quantities{
```

```
  vector[Nind] yrep;
  for (i in 1:Nind){
    yrep[i] = neg_binomial_2_log_rng(b[sp[i], st[i]], phi);
  }
}
```

S5.4 Model results

Estimates, 90% HPDI, Rhat values, effective sample sizes and the proportions of the posterior distribution on the same side of zero as the mean (f) are given. This value (f) represents our confidence that a parameter value is either greater or less than 0.

Table S2: Parameter estimates. Minimum stopover durations per species and departure state are given in days.

	mean	lower	upper	f	rhat	ess_bulk	ess_tail
offshore: Dunnock	11.090	5.411	17.951	1	1.002	4227	2358
offshore: thrushes	17.688	10.552	25.790	1	1.000	3628	2769
offshore: Blackcap	4.189	2.320	6.461	1	1.002	4124	2870
offshore: Garden W.	0.690	0.111	1.616	1	1.002	3208	2732
offshore: Wheatear	4.139	2.325	6.331	1	1.000	4198	2792
onshore: Dunnock	11.509	6.469	17.453	1	1.002	3227	2691
onshore: thrushes	13.008	8.326	18.145	1	1.001	3728	2674
onshore: Blackcap	4.327	2.955	5.892	1	1.000	4337	2873
onshore: Garden W.	3.102	2.124	4.219	1	1.000	3590	2068
onshore: Wheatear	4.370	1.605	7.925	1	1.000	3020	2774
phi	1.279	0.867	1.673	1	1.000	2943	2831

Table S3: Contrasts of minimal stopover duration between departure states in days. Positive values refer to later departures in onshore flights.

	mean	lower	upper	f
Dunnock	0.224	-9.058	9.815	0.534
thrushes	-4.988	-14.483	4.529	0.812
Blackcap	0.034	-2.643	2.735	0.544
Garden W.	2.321	0.901	3.694	0.989
Wheatear	0.517	-3.596	4.361	0.553
mean	0.578	-6.229	7.410	0.660

S6 Departure timing relative to night length

S6.1 Raw data

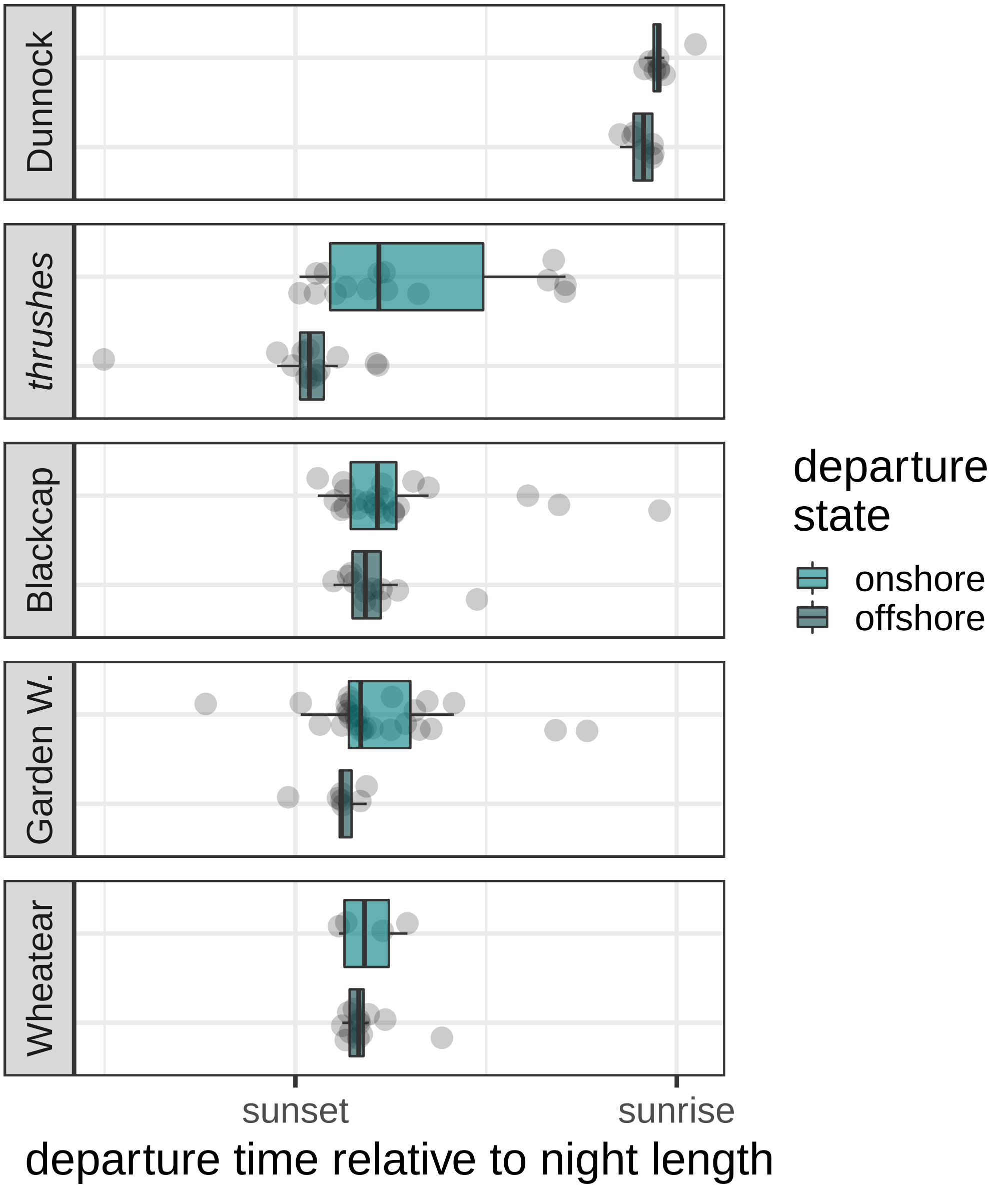

Figure S5: Variation in departure time relative to night length per species and departure state.

S6.2 Model formulation

$$
\begin{aligned}
y_i &\sim \text{Student-}t(v, \mu_i, \sigma) \\
\mu_i &= \beta_{sp[i],st[i]} \\
\begin{bmatrix} \beta_{j,1} \\ \beta_{j,2} \end{bmatrix} &\sim \text{MVNormal}\left(\begin{bmatrix} 0 \\ 0 \end{bmatrix}, S_{sp} \right) \\
S_{sp} &= \begin{pmatrix} \sigma_{\beta[1]} & 0 \\ 0 & \sigma_{\beta[2]} \end{pmatrix} R \begin{pmatrix} \sigma_{\beta[1]} & 0 \\ 0 & \sigma_{\beta[2]} \end{pmatrix} \\
\sigma_{\beta[1]} &\sim \text{Exponential}(1) \\
\sigma_{\beta[2]} &\sim \text{Exponential}(1) \\
R &\sim \text{LKJcorr}(2) \\
v &\sim \text{Exponential}(1) \\
\sigma &\sim \text{Exponential}(1)
\end{aligned}
$$

S6.3 Model code

```
// non-centered parameterisation

data{
  int Nind; // number of individuals
  int  Nsp; // number of species
  vector[Nind] y; // departure relative to night length
  array[Nind] int sp; // species index
  array[Nind] int st; // state index
}

parameters{
  vector<lower=0>[2] sigma_b; // group-level SD
  cholesky_factor_corr[2] L_Rho; // Cholesky correlation factor
  matrix[2,Nsp] z; // z-scores
  real<lower=0> v; // scaling parameter
  real<lower=0> sigma; // population-level SD
}

transformed parameters{
    matrix[Nsp,2] b; // beta
    b = (diag_pre_multiply(sigma_b, L_Rho) * z)';
}

model{
  vector[Nind] mu;

  // Priors and constraints
  sigma_b ~ exponential(1);
  L_Rho   ~ lkj_corr_cholesky(2);
  to_vector(z) ~ normal(0, 1);
  v     ~ exponential(1);
  sigma ~ exponential(1);
  for (i in 1:Nind){
    mu[i] = b[sp[i], st[i]];
  }
```

```
  // Likelihood
  target += student_t_lpdf(y | v, mu, sigma);
}

generated quantities{
  vector[Nind] yrep;
  for (i in 1:Nind){
    yrep[i] = student_t_rng(v, b[sp[i], st[i]], sigma);
  }
}
```

S6.4 Model results

Estimates, 90% HPDI, Rhat values, effective sample sizes and the proportions of the posterior distribution on the same side of zero as the mean (f) are given. This value (f) represents our confidence that a parameter value is either greater or less than 0.

Table S4: Model estimates. Departure timing relative to night length per species and departure state is given together with standard deviation per departure state.

	mean	lower	upper	f	rhat	ess_bulk	ess_tail
offshore: Dunnock	0.907	0.874	0.943	1.000	1.001	4054	2703
offshore: thrushes	0.043	0.013	0.075	0.991	1.001	3080	2648
offshore: Blackcap	0.184	0.149	0.217	1.000	1.000	4515	2815
offshore: Garden W.	0.130	0.093	0.167	1.000	1.000	4043	2830
offshore: Wheatear	0.162	0.136	0.186	1.000	1.001	4356	3264
onshore: Dunnock	0.949	0.919	0.978	1.000	1.002	4051	2818
onshore: thrushes	0.154	0.089	0.220	1.000	1.001	3541	2835
onshore: Blackcap	0.204	0.175	0.235	1.000	1.001	4365	2905
onshore: Garden W.	0.169	0.145	0.194	1.000	1.000	3954	3351
onshore: Wheatear	0.179	0.106	0.260	1.000	1.000	3836	3584
sigma	0.052	0.038	0.066	1.000	1.002	1884	2015
v	1.277	0.875	1.689	1.000	1.002	1969	2128

Table S5: Contrasts between departure states given as the proportion of night length. Positive values refer to later departures or higher variation in onshore flights.

	mean	lower	upper	f
Dunnock	0.042	-0.004	0.085	0.945
thrushes	0.110	0.041	0.186	0.997
Blackcap	0.020	-0.028	0.062	0.776
Garden W.	0.039	-0.005	0.083	0.926
Wheatear	0.018	-0.059	0.103	0.613
mean	0.046	0.019	0.074	0.997

S7 Multistate model

S7.1 Model code

```
data{
  int<lower=0> Nind; // number of individuals
  int<lower=0> Nocc; // number of occasions
  int<lower=0> Nsp;  // number of species
  array[Nind,Nocc] int<lower=1, upper=4> y; // detection history matrix
  array[Nind] int<lower=1, upper=Nsp>   sp; // species index
  array[Nind] int<lower=0, upper=1>    tag; // tag type index

  // Weather data
  matrix[Nind,Nocc] u_l;
  matrix[Nind,Nocc] u_q;
  matrix[Nind,Nocc] v_l;
  matrix[Nind,Nocc] v_q;
  matrix[Nind,Nocc] pc_z;
  matrix[Nind,Nocc] h_z;
  matrix[Nind,Nocc] r;
}

parameters{
  vector<lower=0, upper=1>[Nsp] mean_psi; // mean departure prob. per species
  vector<lower=0, upper=1>[Nsp] mean_chi; // mean prob. for offshore flight per species
  real<lower=0, upper=1>        pX_A;     // mean detection prob. offshore (ACT)
  real<lower=0, upper=1>        pC_A;     // mean detection prob. onshore (ACT)

  // Slope parameters
  vector[Nsp] b_u1_l;
  vector[Nsp] b_u1_q;
  vector[Nsp] b_v1_l;
  vector[Nsp] b_v1_q;
  vector[Nsp] b_p1;
  vector[Nsp] b_h1;
  real b_r1;
  real b_u2_l;
  real b_u2_q;
  real b_v2_l;
  real b_v2_q;
  real<upper=0> eps1; // constrain detection prob. to be lower in NTQB
  real<upper=0> eps2;
}

transformed parameters{
  matrix[Nind,Nocc-1] psi; // departure probability
  matrix[Nind,Nocc-1] chi; // probability for offshore flight
  vector[Nsp]   mu1;       // intercept psi
  vector[Nsp]   mu2;       // intercept chi
  vector[Nind]   pX;       // detection probability offshore
  vector[Nind]   pC;       // detection probability onshore
  real mu_pX;              // intercept pX
  real mu_pC;              // intercept pC
  array[4,Nind,Nocc-1] simplex[4] ps; // state-transition matrix
```

```
array[4,Nind,Nocc-1] simplex[4] po; // observation matrix

// CONSTRAINTS
// Logit transformations
for (s in 1:Nsp){
  mu1[s] = logit(mean_psi[s]);
  mu2[s] = logit(mean_chi[s]);
}
mu_pX = logit(pX_A);
mu_pC = logit(pC_A);

// Linear models
for (i in 1:Nind){
  for (t in 1:(Nocc-1)){
    psi[i,t] = inv_logit(mu1[sp[i]]
    + b_u1_l[sp[i]]*u_l[i,t]  + b_u1_q[sp[i]]*u_q[i,t]
    + b_v1_l[sp[i]]*v_l[i,t]  + b_v1_q[sp[i]]*v_q[i,t]
    + b_p1[sp[i]]  *pc_z[i,t] + b_h1[sp[i]]  *h_z[i,t]
    + b_r1         *r[i,t]);
    chi[i,t] = inv_logit(mu2[sp[i]]
    + b_u2_l*u_l[i,t] + b_u2_q*u_q[i,t]
    + b_v2_l*v_l[i,t] + b_v2_q*v_q[i,t]);
  }
  pX[i] = inv_logit(mu_pX + eps1*tag[i]);
  pC[i] = inv_logit(mu_pC + eps2*tag[i]);
}

// Define state-transition and observation matrices
for (i in 1:Nind){
  for (t in 1:(Nocc-1)){
    // Define probabilities of state S(t+1) given S(t)
    ps[1,i,t,1] = 1-psi[i,t];
    ps[1,i,t,2] = psi[i,t]*chi[i,t];
    ps[1,i,t,3] = psi[i,t]*(1-chi[i,t]);
    ps[1,i,t,4] = 0;
    ps[2,i,t,1] = 0;
    ps[2,i,t,2] = 0;
    ps[2,i,t,3] = 0;
    ps[2,i,t,4] = 1;
    ps[3,i,t,1] = 0;
    ps[3,i,t,2] = 0;
    ps[3,i,t,3] = 0;
    ps[3,i,t,4] = 1;
    ps[4,i,t,1] = 0;
    ps[4,i,t,2] = 0;
    ps[4,i,t,3] = 0;
    ps[4,i,t,4] = 1;

    // Define probabilities of O(t) given S(t)
    po[1,i,t,1] = 1;
    po[1,i,t,2] = 0;
    po[1,i,t,3] = 0;
    po[1,i,t,4] = 0;
```

```
      po[2,i,t,1] = 0;
      po[2,i,t,2] = pX[i];
      po[2,i,t,3] = 0;
      po[2,i,t,4] = 1-pX[i];
      po[3,i,t,1] = 0;
      po[3,i,t,2] = 0;
      po[3,i,t,3] = pC[i];
      po[3,i,t,4] = 1-pC[i];
      po[4,i,t,1] = 0;
      po[4,i,t,2] = 0;
      po[4,i,t,3] = 0;
      po[4,i,t,4] = 1;
    }
  }
}

model{
  array[4] real acc; // accumulator
  array[Nocc,4] real gam; // forward values

  // PRIORS
  b_u1_l ~ normal(0, 10);
  b_u1_q ~ normal(0, 10);
  b_v1_l ~ normal(0, 10);
  b_v1_q ~ normal(0, 10);
  b_p1   ~ normal(0, 5);
  b_h1   ~ normal(0, 5);
  b_r1   ~ normal(0, 5);
  b_u2_l ~ normal(0, 10);
  b_u2_q ~ normal(0, 10);
  b_v2_l ~ normal(0, 10);
  b_v2_q ~ normal(0, 10);

  // LIKELIHOOD
  // Forward algorithm derived from Stan
  // user's guide and reference manual
  for (i in 1:Nind){
    // Make sure that all individuals are in state 1 at t=1
    gam[1,1] = 1;
    gam[1,2] = 0;
    gam[1,3] = 0;
    gam[1,4] = 0;

    for (t in 2:Nocc){
      for (k in 1:4){   // current state (t)
        for (j in 1:4){ // previous state (t-1)
          acc[j] = gam[t-1,j] * ps[j,i,t-1,k]
                   * po[k, i, t-1, y[i,t]];
        }
        gam[t,k] = sum(acc);
      }
    }
    target += log(sum(gam[Nocc]));
```

```
  }
}

generated quantities{
  array[Nind,Nocc] int<lower=1, upper=4> z; // latent state
  int  Noff; // number of offshore flights
  real Nrel; // proportion of offshore flights

  // Generate z[]
  for (i in 1:Nind){
    z[i,1] = 1;
    for (t in 2:Nocc){
      z[i,t] = categorical_rng(ps[z[i,t-1], i, t-1]);
    }
  }

  // Count offshore flights
  Noff = 0;
  for (i in 1:Nind){
    for (t in 1:Nocc){
      if (z[i,t] == 2){
        Noff += 1;
      }
    }
  }
  Nrel = Noff*1.0 / Nind;

  // Calculate detection probabilities for NTQB tags
  real pX_N = inv_logit(mu_pX + eps1);
  real pC_N = inv_logit(mu_pC + eps2);
}
```

S7.2 Model results

Estimates, 90% HPDI, Rhat values, effective sample sizes and the proportions of the posterior distribution on the same side of zero as the mean (f) are given. This value (f) represents our confidence that a parameter value is either greater or less than 0.

Table S6: Species-specific influence of weather on departure decision, i.e. the day-to-day departure probability (Log Odds).

	mean	lower	upper	f	rhat	ess_bulk	ess_tail
intercept: Dunnock	-2.431	-3.007	-1.892	1.000	1.002	2813	2510
intercept: thrushes	-2.533	-2.791	-2.276	1.000	1.000	3328	2850
intercept: Blackcap	-1.230	-1.543	-0.934	1.000	1.000	3190	2951
intercept: Garden W.	-0.522	-0.916	-0.160	0.985	1.001	2841	2336
intercept: Wheatear	-1.731	-2.233	-1.162	1.000	1.001	2799	2467
eastward wind (linear): Dunnock	6.401	-8.934	21.791	0.751	1.001	3818	2708
eastward wind (linear): thrushes	9.614	-4.005	22.640	0.875	1.002	3697	2664
eastward wind (linear): Blackcap	4.284	-9.945	18.290	0.696	0.999	4135	2989
eastward wind (linear): Garden W.	3.095	-12.328	19.137	0.630	1.000	3398	2791
eastward wind (linear): Wheatear	-7.191	-23.293	8.740	0.769	1.000	4382	2755
eastward wind (quadratic): Dunnock	-5.548	-20.887	9.169	0.721	1.001	3992	2755
eastward wind (quadratic): thrushes	-12.038	-24.076	-0.172	0.955	1.002	4262	2915
eastward wind (quadratic): Blackcap	-11.435	-25.609	2.360	0.913	1.001	3825	2237
eastward wind (quadratic): Garden W.	1.572	-12.897	16.774	0.568	1.001	4239	2607
eastward wind (quadratic): Wheatear	4.059	-10.798	20.010	0.665	1.000	3887	2574
northward wind (linear): Dunnock	5.994	-10.770	21.706	0.723	1.000	3526	2924
northward wind (linear): thrushes	7.251	-7.142	20.809	0.797	1.000	3454	2695
northward wind (linear): Blackcap	7.872	-6.484	22.538	0.812	1.001	3407	2281
northward wind (linear): Garden W.	11.216	-4.162	24.892	0.898	1.002	3927	2669
northward wind (linear): Wheatear	7.370	-7.044	22.059	0.794	1.006	3976	2799
northward wind (quadratic): Dunnock	8.267	-6.845	25.775	0.804	1.000	3780	2655
northward wind (quadratic): thrushes	4.764	-9.632	19.769	0.702	1.001	3788	2755
northward wind (quadratic): Blackcap	2.111	-12.305	16.372	0.590	1.000	4130	2627
northward wind (quadratic): Garden W.	-10.045	-23.122	3.459	0.897	1.001	3298	2673
northward wind (quadratic): Wheatear	-8.326	-23.353	7.197	0.820	1.001	4402	2789
pressure change: Dunnock	0.053	-0.348	0.467	0.580	1.000	3332	2548
pressure change: thrushes	0.360	0.136	0.583	0.996	1.001	3033	3128
pressure change: Blackcap	0.004	-0.295	0.332	0.505	1.001	3650	2665
pressure change: Garden W.	-0.029	-0.489	0.435	0.539	1.003	3054	2600
pressure change: Wheatear	-0.332	-0.701	0.032	0.933	1.002	4108	2780
relative humidity: Dunnock	0.095	-0.467	0.672	0.586	1.000	2844	2446
relative humidity: thrushes	-0.442	-0.711	-0.187	0.998	1.000	3542	2332
relative humidity: Blackcap	-0.173	-0.477	0.109	0.833	1.002	3104	2859
relative humidity: Garden W.	0.044	-0.378	0.482	0.566	1.001	3117	3031
relative humidity: Wheatear	-0.901	-1.426	-0.353	0.998	1.001	2868	2633
precipitation (rain)	-1.368	-2.079	-0.704	1.000	1.001	3281	2403

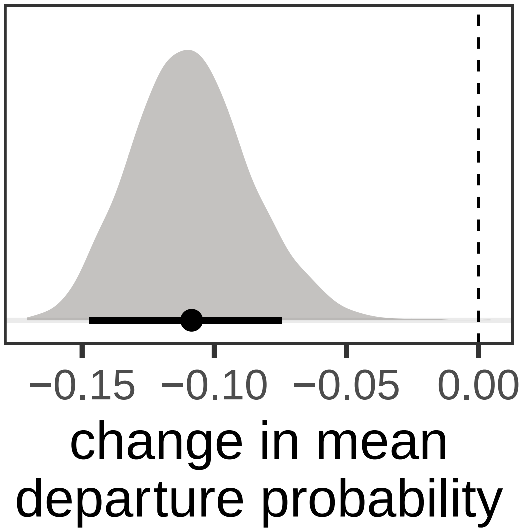

Figure S6: Influence of precipitation on departure decision, i.e. the day-to-day departure probability, from stopover during spring. The posterior distribution for the contrast between rain and no rain is given.

Table S7: Influence of wind on routing decision, i.e. the probability for an offshore flight (Log Odds).

	mean	lower	upper	f	rhat	ess_bulk	ess_tail
intercept: Dunnock	-0.103	-0.940	0.724	0.577	1.001	3989	2491
intercept: thrushes	0.803	0.240	1.299	0.994	1.000	3389	2916
intercept: Blackcap	-0.042	-0.551	0.468	0.543	1.000	3486	2314
intercept: Garden W.	-0.925	-1.559	-0.240	0.995	1.002	3808	2671
intercept: Wheatear	1.526	0.658	2.447	0.999	1.001	3669	2270
eastward wind (linear)	-7.654	-22.051	6.069	0.818	1.002	3676	2812
eastward wind (quadratic)	-5.720	-19.699	8.496	0.741	1.001	4773	2285
northward wind (linear)	8.192	-6.055	21.965	0.835	1.002	3945	2745
northward wind (quadratic)	-2.573	-16.280	11.244	0.610	1.000	3853	2738

Table S8: Species-specific departure probabilities (psi) and probabilities for offshore flights (chi) together with tag-specific detection probabilities for offshore (pX) and onshore (pC) flights. Additionally, the estimated number and proportion of offshore flights are given.

	mean	lower	upper	f	rhat	ess_bulk	ess_tail
psi: Dunnock	0.084	0.044	0.126	1	1.002	2813	2510
psi: thrushes	0.074	0.057	0.092	1	1.000	3328	2850
psi: Blackcap	0.228	0.176	0.282	1	1.000	3190	2951
psi: Garden W.	0.374	0.286	0.460	1	1.001	2841	2336
psi: Wheatear	0.155	0.089	0.224	1	1.001	2799	2467
chi: Dunnock	0.476	0.281	0.674	1	1.001	3989	2491
chi: thrushes	0.687	0.575	0.798	1	1.000	3389	2916
chi: Blackcap	0.490	0.366	0.615	1	1.000	3486	2314
chi: Garden W.	0.291	0.153	0.414	1	1.002	3808	2671
chi: Wheatear	0.809	0.692	0.942	1	1.001	3669	2270
pX: ACT	0.498	0.402	0.585	1	1.001	3173	2343
pX: NTQB	0.461	0.375	0.546	1	1.002	4338	3215
pC: ACT	0.966	0.914	1.000	1	1.001	1748	1358
pC: NTQB	0.940	0.871	1.000	1	1.000	2449	2096
number of offshore flights	99.002	83.000	113.000	1	1.000	3358	2946
proportion of offshore flights	0.538	0.451	0.614	1	1.000	3358	2946

S7.3 Probabilities for offshore flights in birds with unknown routing

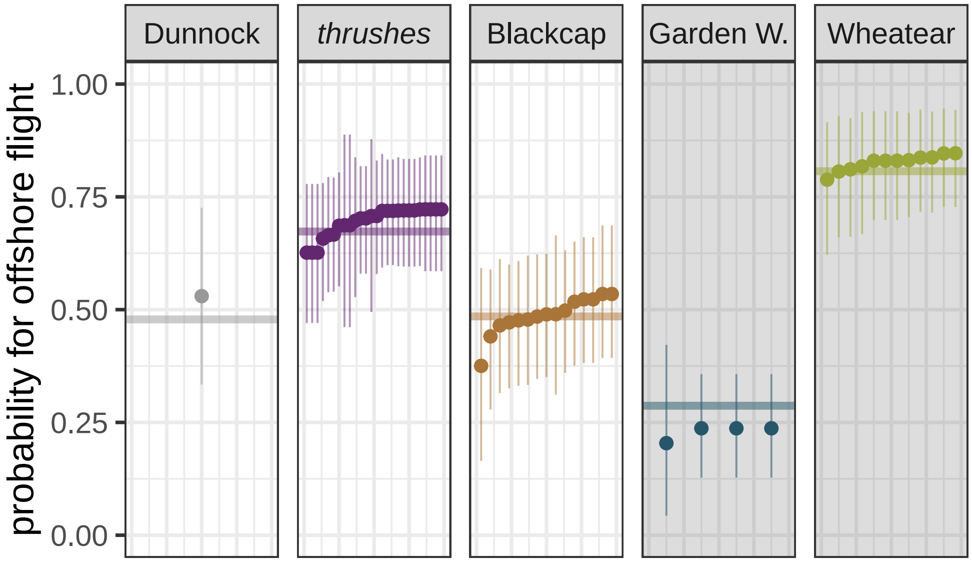

Figure S7: Predicted probabilities for offshore flights in birds with unknown routing. Predictions (dots) and 90% HPDI (segments) are given. Horizontal lines represent the estimated mean probability per species.

S8 Further potential effects on routing

We used binary regression models and the package `brms` to assess the relationships between routing decision and intrinsic factors, i.e. age, sex and weight.

S8.1 Effect of age and sex

```
# load packages
library(dplyr)
library(ggplot2)
library(brms)
library(HDInterval)

# helper function
p_probs_brm <- function(mod = NULL) {
  bsim <- fixef(mod, summary = F)
  p.probs <- data.frame(f = rep(NA, ncol(bsim)), row.names = colnames(bsim))
  for (i in 1:ncol(bsim)) {
    p.probs[i,] <- round(ifelse(mean(bsim[,i]) > 0,
                                sum(bsim[,i] > 0),
                                sum(bsim[,i] < 0)) / nrow(bsim), 2)
  }
  return(p.probs)
}

# prepare data
tmp <- mod.data %>%
  filter(flightCat != "departed") %>%
```

```r
  mutate(flightCat = ifelse(flightCat == "onshore", 0, 1))
tmp$age[tmp$age == "> 1st year"] <- NA

# model
l1 <- brm(flightCat ~ age + sex, data = tmp, family = bernoulli(),
          backend = "cmdstanr", refresh = 0)
```

```
## Running MCMC with 4 sequential chains...
##
## Chain 1 finished in 0.1 seconds.
## Chain 2 finished in 0.1 seconds.
## Chain 3 finished in 0.1 seconds.
## Chain 4 finished in 0.0 seconds.
##
## All 4 chains finished successfully.
## Mean chain execution time: 0.1 seconds.
## Total execution time: 0.6 seconds.
```

```r
mcmc_plot(l1, type = "areas")
```

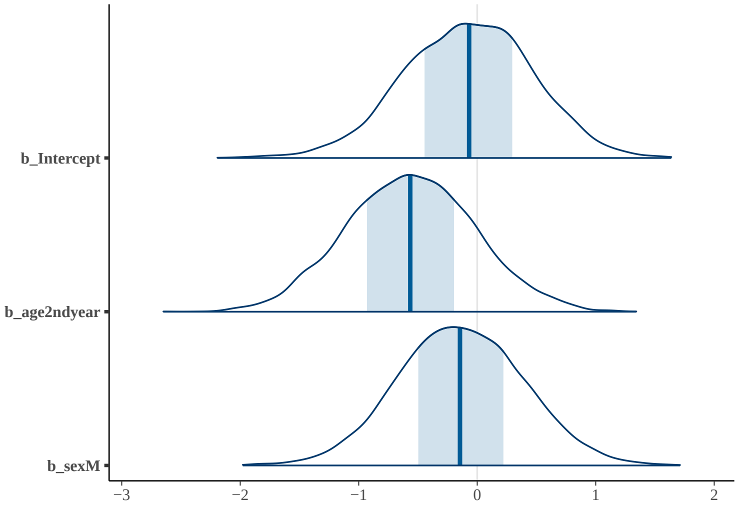


```r
# posterior probabilities
p_probs_brm(l1)
```

```
##               f
## Intercept  0.55
## age2ndyear 0.85
## sexM       0.60
```

```r
# effect plot age
newdat <- data.frame(age = c("2nd year", "> 2nd year"),
                     sex = "F")
pp <- fitted(l1, newdata = newdat, re_formula = NA, summary = F)
contrast <- pp[,1] - pp[,2]

g <- ggplot() +
  tidybayes::stat_halfeye(aes(contrast), point_interval = "mean_hdi",
                          .width = 0.9) + ylab(NULL) +
  geom_vline(xintercept = 0, lty = 2) + theme_bw(base_size = 20)
```

```
g
```

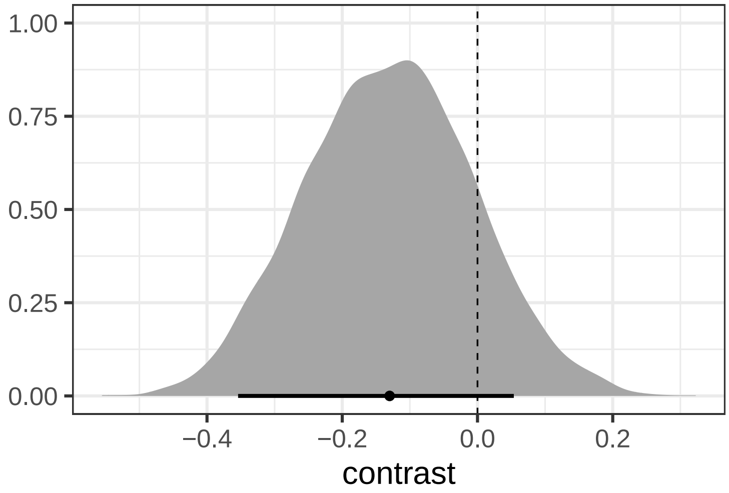

S8.2 Effect of weight

Since weight only is known for the first day, i.e. tag deployment, we only included birds that departed within the first night after release. Blackcaps (15) and Garden Warblers (16) had sufficient sample sizes. We corrected weight by wing length and scales it per species.

```
# prepare data
tmp <- tmp %>%
  filter(min_stopover == 0 & speciesEN != "Wheatear") %>%
  group_by(speciesEN) %>%
  mutate(w = scale(weight/wing))

# model
l2 <- brm(flightCat ~ w, data = tmp, family = bernoulli(),
          backend = "cmdstanr", refresh = 0)
```

```
## Start sampling
```

```
## Running MCMC with 4 sequential chains...
##
## Chain 1 finished in 0.1 seconds.
## Chain 2 finished in 0.1 seconds.
## Chain 3 finished in 0.1 seconds.
## Chain 4 finished in 0.1 seconds.
##
## All 4 chains finished successfully.
## Mean chain execution time: 0.1 seconds.
## Total execution time: 0.6 seconds.
```

```
mcmc_plot(l2, type = "areas")
```

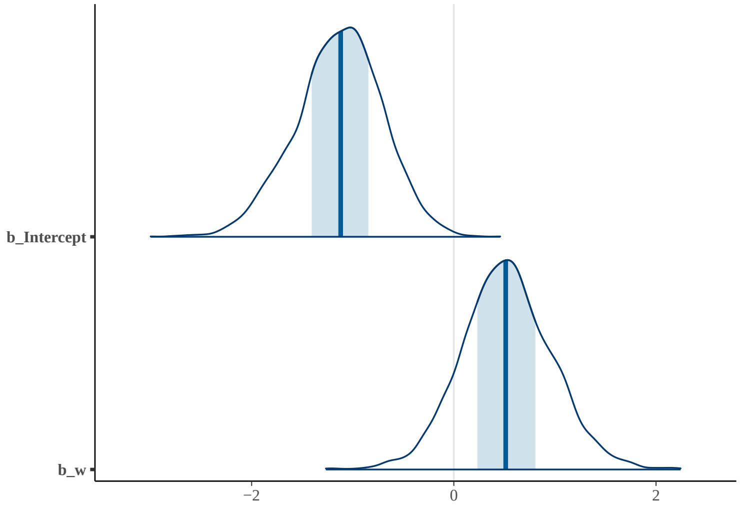


```
# posterior probabilities
p_probs_brm(l2)
```

```
##              f
## Intercept 1.00
## w         0.89
```

```
# effect plot
newdat <- data.frame(w = seq(min(tmp$w), max(tmp$w), length = 100))
pp     <- fitted(l2, newdata = newdat, re_formula = NA, summary = F)
sim    <- get_sims(newdat$w, pp, 1)

g <- ggplot() +
  geom_line(aes(x, sim, group = draw), data = sim, alpha = 0.2) +
  geom_line(aes(newdat$w, colMeans(pp)), lwd = 2) + ylim(0, 1) +
  labs(x = "weight (scaled)", y = "probability for offshore flight") +
  theme_bw(base_size = 20)

g
```

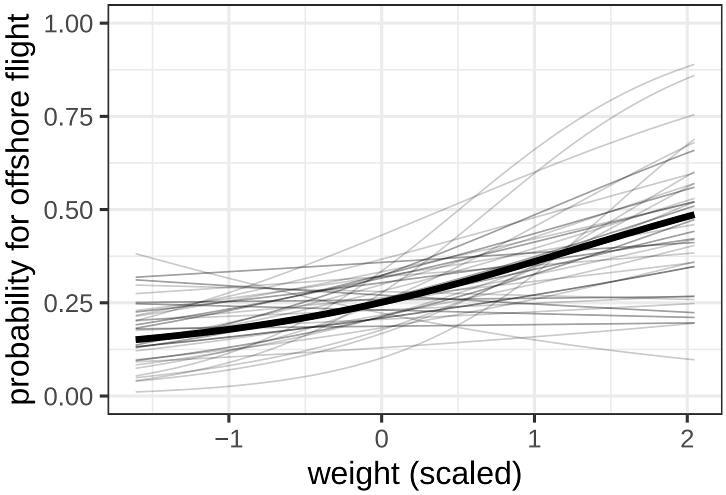

S9 Wind drift

To get an idea to which extend radio-tagged birds were affected by wind drift, we estimated flight bearings in respect to wind direction *wdir* using a sinusoidal model including parameters for intercept α, phase *pha*, and amplitude *amp* in which we weighted the log likelihood by the squared wind speed *wsp*.

$$\sum_{n=1}^{N} wsp_i^2 \text{ Log normal}(y_i|\mu_i, \sigma)$$

$$\mu_i = \alpha + \sin(wdir_i + pha)\ amp$$

```
data{
  int<lower=0> N;  // number of individuals
  vector[N] y;     // individual bearings
  vector[N] wdir;  // wind direction
  vector[N] wsp;   // wind speed
}

parameters{
  real a;
  real<lower=0, upper=2*pi()> pha; // phase
  real<lower=0> amp;   // amplitude
  real<lower=0> sigma; // sd
}

transformed parameters{
  vector[N] mu = a + sin(wdir+pha)*amp;
}

model{
  for (i in 1:N){
    target += wsp[i]^2 * normal_lpdf(y[i] | mu[i], sigma); // weighted likelihood
  }
}
```

We found some indication of wind drift in our data, i.e. birds' flight bearings were shifted northward under SE cross winds and southward under NW cross winds. However, given the total distribution of wind directions, birds must have been able to compensate for wind drift.

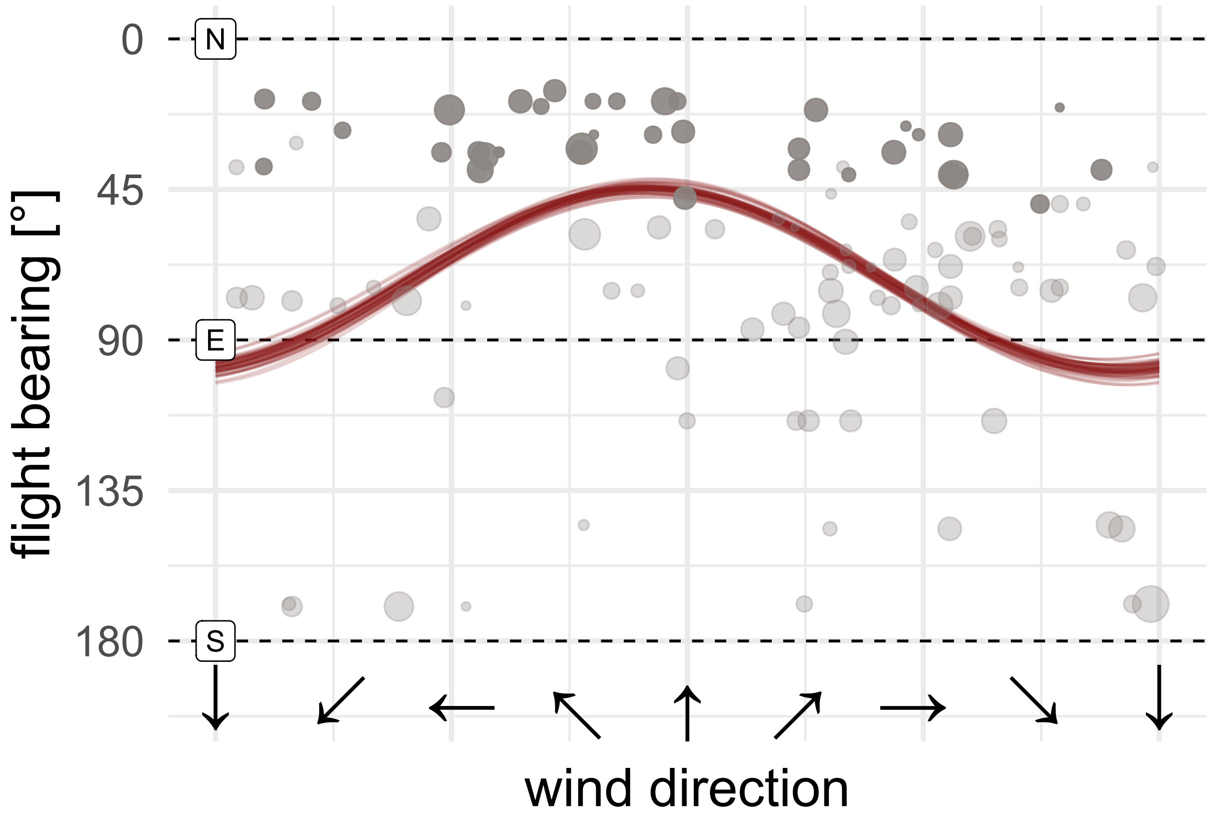

Figure S8: Flight bearings of individual offshore (dark grey points) and onshore flights (light grey points) plotted against wind direction. West-oriented flights and flights including detections on Helgoland were excluded from the analysis. Point sizes refer to the squared wind speed. Lines represent a subset of 50 draws from the joint posterior distribution of the sinusoidal model.
